# Supplementary material for: Inhibitory Investigations of Acyl-CoA Derivatives against Human Lipoxygenase Isozymes
Source: Int J Mol Sci. 2023 Jun 30;24(13):10941. doi: 10.3390/ijms241310941 (PMC10341549; doi:10.3390/ijms241310941)
Supplement: Supplementary file 1 [file ijms-24-10941-s001.zip › ijms-2462870-supplementary.pdf]

SUPPORTING INFORMATION

Inhibitory Investigations of Acyl-CoA Derivatives against Human Lipoxygenase Isozymes

Michelle Tran,[1] Kevin Yang[1], Alisa Glukhova,[2] Michael Holinstat,[3] Theodore Holman[1]*

[1]Department of Chemistry and Biochemistry, University of California Santa Cruz, Santa Cruz, CA 95064, United States

[2] Department of Biochemistry and Pharmacology, University of Melbourne, Melbourne, Victoria, Australia

[3] Department of Pharmacology, University of Michigan Medical School, Ann Arbor, MI, 48109

Funding: NIH-GM131835

***Corresponding Author:**

TRH: Tel.: +1-831-459-5884, holman@ucsc.edu

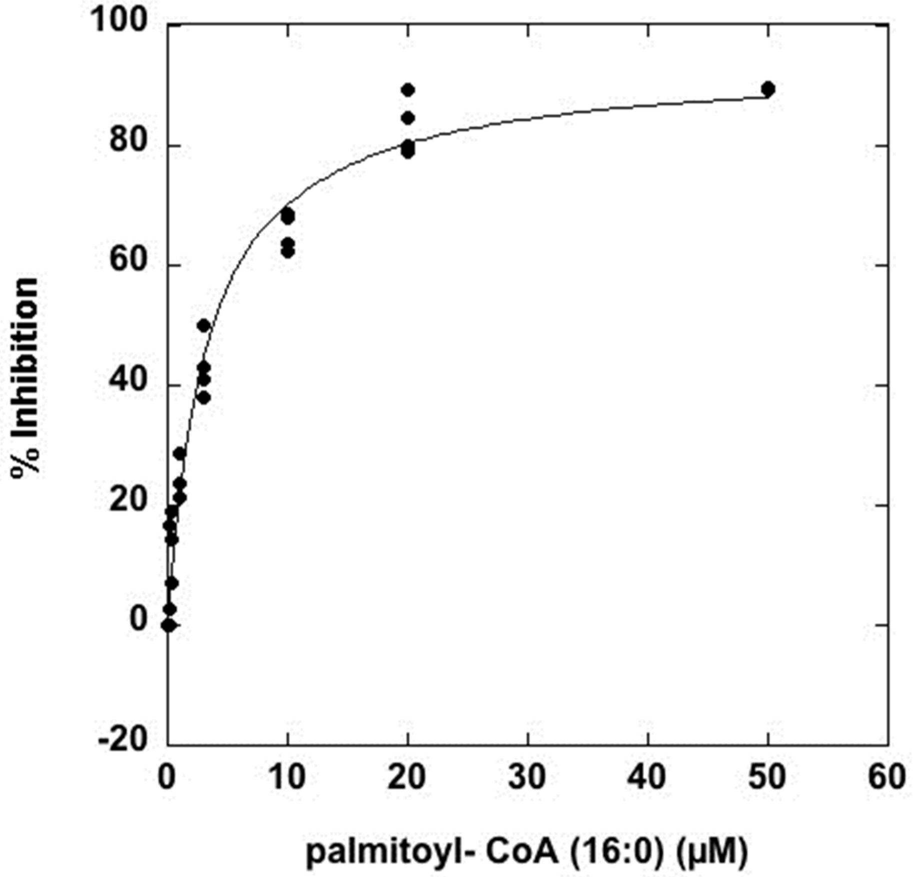

Supplemental Figure S1. IC_{50} of Palmitoyl-CoA (16:0) with h5-LOX

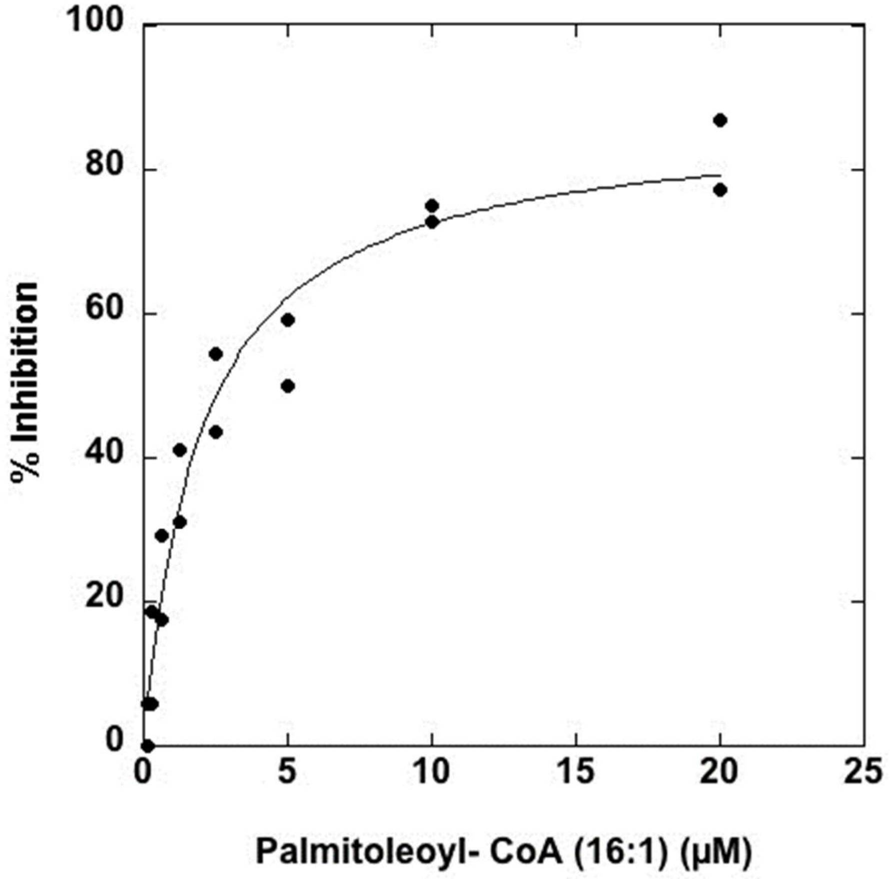

Supplemental Figure S2. IC_{50} of Palmitoleoyl-CoA (16:1) with h5-LOX

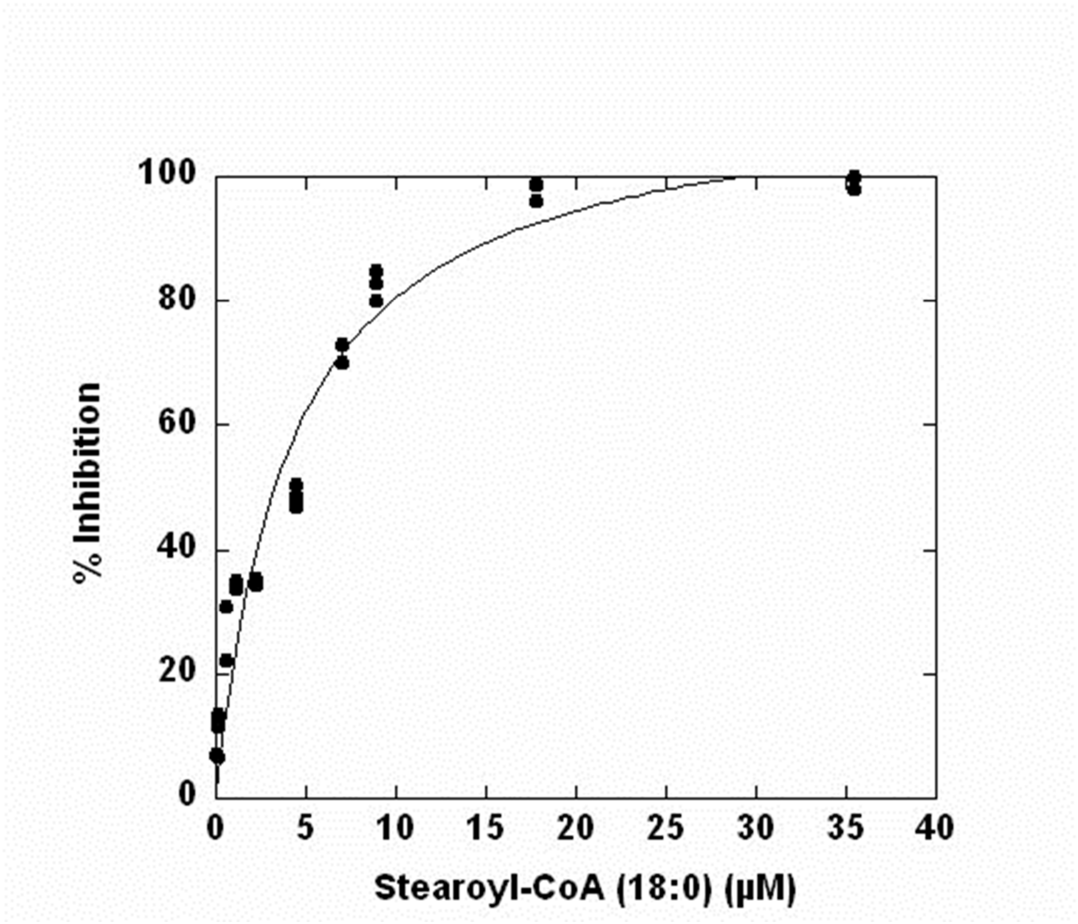

Supplemental Figure S3. IC_{50} of the h15-LOX-1 with Stearoyl-CoA (18:0)

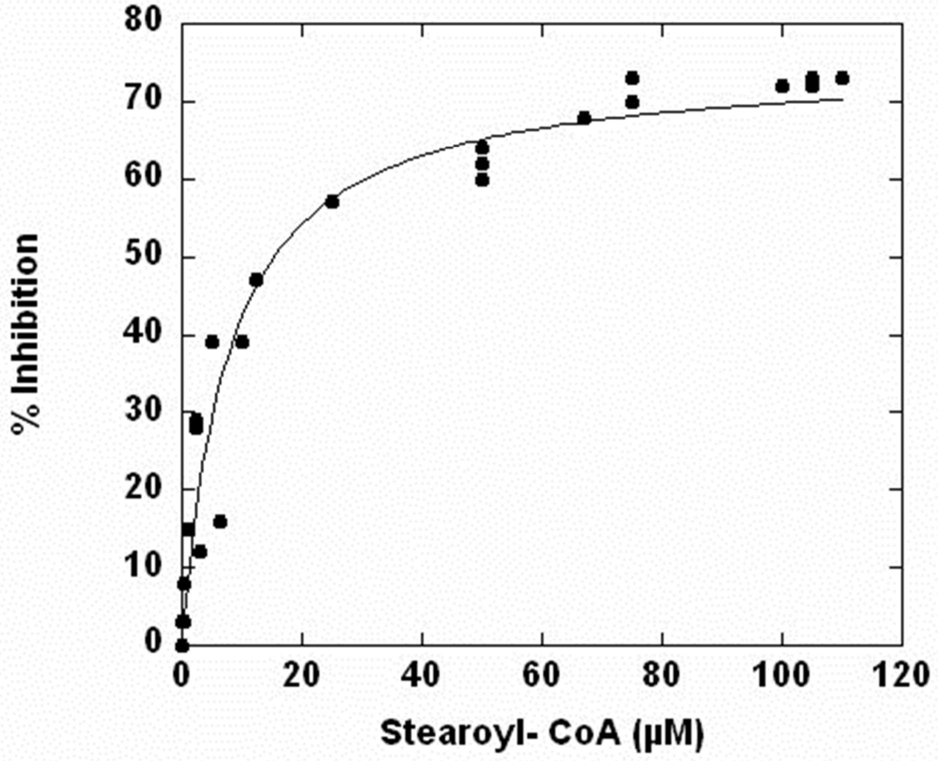

Supplemental Figure S4. IC_{50} of h15-LOX-2 with Stearoyl-CoA(18:0)

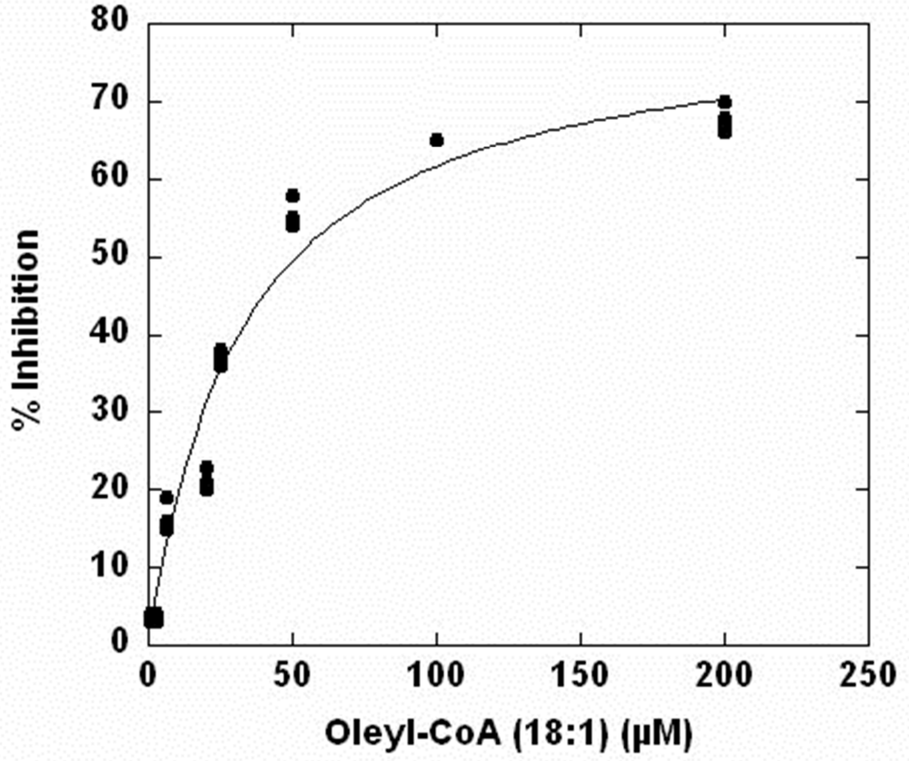

Supplemental Figure S5. IC_{50} of the Oleoyl-CoA (18:1) with h12-LOX

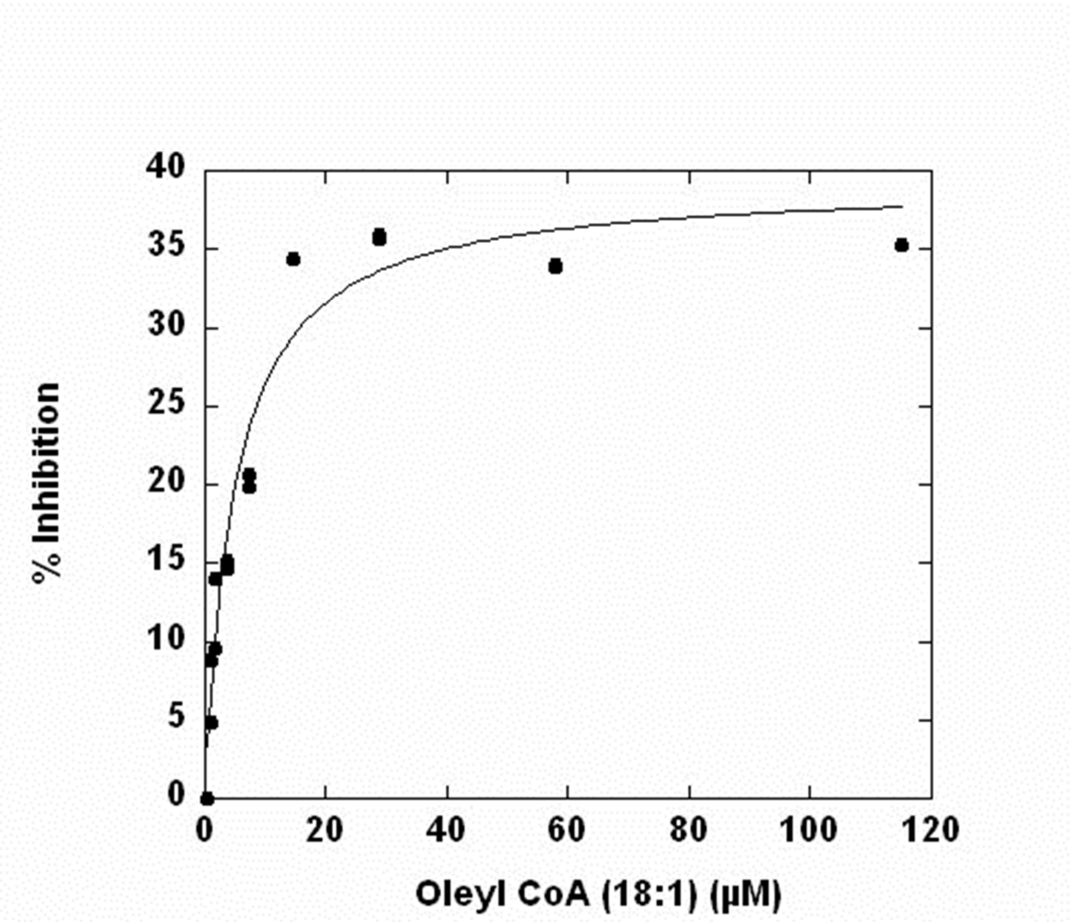

Supplemental Figure S6. IC_{50} of Oleoyl-CoA (18:1) with h15-LOX-1

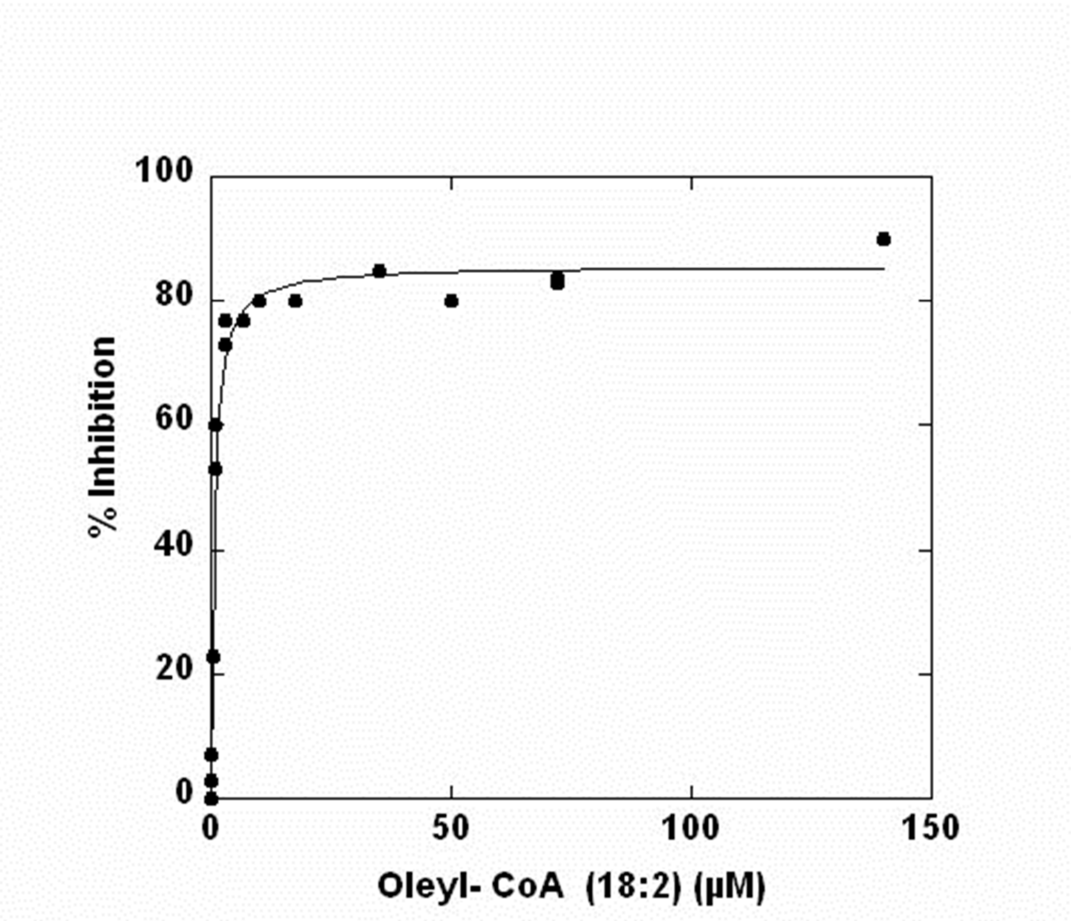

Supplemental Figure S7. IC_{50} of Oleoyl-CoA (18:1) with h15-LOX-2

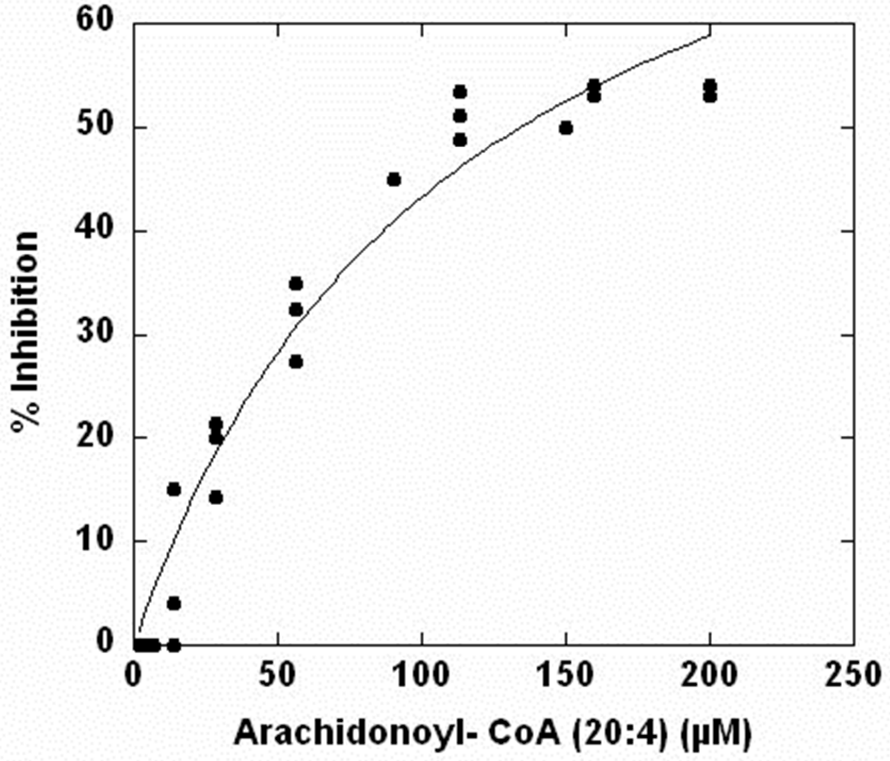

Supplemental Figure S8. IC_{50} of Arachidonoyl-CoA (20:4) with h12-LOX
